# Clinical, Microbiological, and Microcomputed Tomography Evaluation of Silver Diamine Fluoride in Controlling Root Carious Lesions: An *in Vivo* Study

**DOI:** 10.4317/jced.61178

**Published:** 2024-07-01

**Authors:** Natnicha Chitpitak, Paweena Wongwitwichot, Supitcha Talungchit, Supawadee Naorungroj

**Affiliations:** 1 Dental Public Health Department, Sribunpot Hospital, Phatthalung, Thailand; 2 Department of Pharmaceutical Chemistry, Faculty of Pharmaceutical Sciences, Prince of Songkla University, Hat Yai, Songkhla, Thailand; 3 Department of Conservative Dentistry, Faculty of Dentistry, Prince of Songkla University, Hat Yai, Songkhla, Thailand; 4 Research Center of Excellence for Oral Health, Faculty of Dentistry, Prince of Songkla University, Songkhla, Thailand

## Abstract

**Background:**

Silver diamine fluoride (SDF) application without removing necrotic tissue is an applicable non-invasive measure to primary care practice and may reduce the burden of untreated root caries. This study aims to examine clinical feature change, root caries-related bacteria, and silver penetration of SDF in arresting root caries.

**Material and Methods:**

Ten study participants with 16 root carious teeth were included in this study. The clinical characteristics of root caries lesions (plaque deposit, color, hardness, and sensitivity symptom) were recorded. Then root caries samples were collected using a spoon excavator before and 2 weeks after treated with 38% SDF. The amounts of *Streptococcus mutans* (*S.mutans*), *Actinomyces naeslundii* (*A. naeslundii*), and *Lactobacillus casei* (*L. casei*) were determined using real-time PCR. Any tooth sample scheduled for extraction was further analyzed using micro-CT, stereoscopic microscope, and FE-SEM/ EDX to determine the silver penetration.

**Results:**

Most treated samples were darker in color, predominantly turning black (n =15, 93.8%), had increased surface hardness (n =11, 68.8%), were non-sensitive teeth (n=14, 87.5%), and were negative to air blowing (n =12, 75%). Only *S.mutans* had a significantly lower number of bacteria after 2 weeks (*p*-value = 0.041). The micro-CT analysis revealed that the silver increased the root carious lesion’s density in proportion to its depth. According to a stereoscopic microscope study, silver penetration caused dark bands, appearing along the dentinal tubule toward the dental pulp. An FE-SEM analysis showed that silver was found to be densely deposited on the surface of the lesions and penetrated through the dentinal tubule into the dental pulp direction. EDX mapping confirmed that the increased density was related to silver.

**Conclusions:**

Based on clinical and microbiological profiles, this investigation indicated that SDF is beneficial for controlling root caries, particularly *S.mutans* reduction. Silver can also penetrate deep into the lesion.

** Key words:**Microbiology, Root caries, Silver diamine fluoride, Silver ion, Streptococcus mutans, Actinomyces naeslundii, Lactobacillus casei.

## Introduction

The preservation and maintenance of dentition throughout life is a fundamental goal of the dental profession. Older adults are more vulnerable to root caries and periodontal disease, which cause tooth loss in this age group (1-4). Both conditions also cause pain and impact quality of life (4). In community dwellings, the prevalence of root caries ranged from 8% to 74% (2). Diverse socioeconomic backgrounds along with clinical and behavioral risk factors, have been linked to root caries. High-risk factors encompass older age, lower socioeconomic status, poor oral hygiene, hyposalivary functions, physical and mental disabilities, the presence of dentures, and gingival recession (5).

While a study conducted among community-dwelling elders supported the repeated application of 38% silver diamine fluoride SDF at 6-month intervals for the arrest of root caries (6), other investigations have recommended an annual application of either SDF solution or a combination of SDF and potassium iodide (KI) solution to halt the progression of dental root caries in older adults (7-9). Furthermore, a systematic review with meta-analysis also supported that among the professionally applied topical fluorides, an annually applied 38% SDF solution in conjunction with oral health education is the most promising non-invasive treatment for preventing dental root caries (10).

Not only clinical evidence, but there is also an effort to investigate the mechanisms of action of SDF in caries-arresting efficiency, particularly its antibacterial effects on mono-cariogenic species biofilm (11-13), multi-cariogenic species biofilm (14), or an even more complex approach, metagenomics (15-17). Caries is a disease process involving multiple factors in which the primary oral pathogen responsible for coronal and root caries is *Streptococcus mutans* (*S.mutans*). In addition to *S.mutans*, Actinomyces, *Lactobacilli*, *Atopobium, Olsenella, Pseudoramibacter, Propionibacterium*, and *Selenomonas* and species also have been reported as putative etiologic factors for root caries (18,19). Due to the complexity of the biofilm environment in root caries (18,19), *in vitro* biofilm investigations are difficult to replicate the microbial environment in the mouth cavity. However, few studies have been quantitatively investigated the effect of SDF treatment on microbial burden for root caries (12,13,17) and related this change to the clinical evidence as well as silver penetration.

Accordingly, this present study aims to examine the short-term effectiveness of SDF in controlling of root carious lesions by (i) evaluation of clinical feature changes of color, hardness, tooth hypersensitivity, an plaque accumulation; (ii) quantitative evaluation of three root caries-associated microbes using Real-time PCR; (iii) evaluation of silver penetration using Micro-computed tomography (micro-CT), Stereoscopic microscope, and Scanning electron microscope with Energy Dispersive X-ray Spectrometer (FE-SEM/EDX).

## Material and Methods

-Study design and study participants

This study protocol was approved by the Institutional Review Board of the Faculty of Dentistry, Prince of Songkla University (EC6305-017) and registered to Thai Clinical Trials Registry (TCTR20211110003). This study employed a quasi-experimental design (pre-post intervention, non-randomized) in which the intervention was administered in a clinic setting.

Sample size calculation:

In order to determine the sample size, we employed the Wilcoxon signed-rank test as the statistical method for analysis, due to its appropriateness for non-parametric data and paired observations, using the G*Power Version 3.1.9.4 software. The sample size for this study was determined based on the primary outcome measure, which was the effectiveness of SDF in reducing the amount of root caries-associated bacteria in log colony-forming units (CFU) before and 2 weeks after treatment. The anticipated average decrease in bacterial count was at least 1.5 log CFU with a standard deviation of 1 which is equivalent to an effect size of 1.5. The study was conducted with a statistical power of 0.80, a significance level (alpha) of 0.05, and a two-tailed test. Based on the assumptions, the desired sample size was 6. With an estimated 25% dropout rate, it was necessary to recruit roughly 8 individuals at the beginning of the study.

Participants, inclusion Criteria, and setting.

Ten (n = 10) adults aged 40 years and older who met the following inclusion criteria were invited to participate in the study: had no communication or cognitive problems; presence of at least one supragingival root carious lesion, lesions have an extension beyond the cemento-enamel junction ≤ 2 mm, and no history of spontaneous pain. Adults whose salivary gland function had been significantly affected by disease, medication, or treatment in the head and neck region such as radiotherapy were excluded. Those with a known sensitivity to silver or other heavy metal were also excluded. Following study consent, a total of 16 root carious samples were included, with three samples scheduled for extraction due to poor periodontal prognosis and pre-prosthodontic reasons. The extracted teeth samples were further under evaluation of silver penetration potential and pattern.

-Baseline data collection

Interview.

During the first visit, study participants were interviewed using a structured questionnaire to collect general information and oral health care information, including tooth sensitivity symptom, brushing frequency, use of fluoride toothpaste, and adjunctive tooth cleaning aids.

Clinical examination and sample collection:

The baseline examination was then performed on a dental chair unit by trained examiners. Periapical status was examined with periapical radiographs. The plaque on the root surface was cleaned with pumice, rinsed thoroughly with water, and dried. The root caries examination was based on visual-tactile examination using periodontal probe. The following clinical characteristics were recorded: visible plaque deposit (0 = no plaque detected; 1 = visible plaque detected); color (yellow, light brown, dark brown, and black); and hardness (hard vs. soft or leathery). If a lesion was soft on probing, it was classified as active (15,20). Tooth sensitivity when gently air blowing was tested. Then, root caries samples containing soft and infected tissue were collected with a sterile spoon excavator and placed into the Tris-EDTA (TE) buffer contained in Eppendorf immediately. Graduate and undergraduate students in the Operative Clinic completed the examinations under the supervision of certified specialists (SP to ST).

Intervention:

For each participant, the clinical procedure was that exposed gingiva and mucosa was protected with petroleum jelly and the carious lesion was isolated with cotton roll and dried with triple syringe. Then, a disposable micro brush was dipped into a drop of 38% SDF (Topamine, 25% w/v silver ion; Dentalife, Melbourne, AUS) and applied to the carious lesion for 60 seconds. The SDF-treated lesion was air-dried gently, and any excess was removed using cotton pellets if necessary (21).

-Follow-up

After 2 weeks, the clinical examination and tooth hypersensitivity test were repeated. Carious lesion sample collection for microbial analysis was carried out by the same examiners using the same equipment. For teeth scheduled for extraction were completed in the same day, and the teeth samples were kept in 4% paraformaldehyde or further study to determine the mineral dentistry using micro-CT. Afterward, tooth samples were prepared for analysis using a stereoscopic microscope and FE-SEM/EDX to identify the pattern of silver penetration.

-Laboratory examination

Real-time PCR:

Isolation of microbial genomic DNA sample: Genomic DNA from microbial culture and root caries samples were extracted using TIANamp Bacteria DNA Kit (TIANGEN Biotech) according to the manufacturers’ protocols. The samples taken from root caries were accurately weighed at 0.0025 grams for extraction, and the amount of extracted DNA was quantified spectrophotometrically.

Quantitative evaluation of microbes in root caries using RT-PCR: Detection and amplification of bacterial DNA were performed with QuantstudioTM 5 Real-Time PCR system (Thermo Fisher Scientific) using Maxima SYBR Green/ROX qPCR Master Mix (2x) (Thermo Fisher Scientific). The reaction consisted of 1x SYBR Green/ROX qPCR Master Mix, 300 nM each of forward and reverse primers, and 2-3 µL of extracted DNA samples. The reaction conditions were pre-treatment at 50°C for 2 minutes, initial denaturation at 95°C for 10 minutes, followed by 40 cycles of denaturation at 95°C for 20 seconds, annealing for 20 seconds and extension at 72°C for 25 seconds. Melting curve analysis was performed at the end of the amplification cycle. The primer sequences and annealing temperature were shown in  Table 1 (22-24). Quantitative RT-PCR was determined in triplicated. The bacterial quantities (log CFU/g) were calculated using standard curve plotted between cycle threshold (CT) values obtained from amplification of known amount of each targeting bacteria (log CFU/mL).

Micro-CT:

Extracted teeth sample that stored in 4% paraformaldehyde were rinsed for 30 minutes. Then, each sample was placed horizontally with the lesion surface facing upward and mounted with dental plaster in a 37 mm diameter by 25 mm height PVC pipe. The scan axis was perpendicular to the direction of dentinal tubule. A clear acrylic sheet was then placed over the PVC pipe and marked with gutta percha with 0.1 mm in diameter (the explorer’s tip), indicating the region of interest (ROI). Each sample was scanned using micro-CT scanner (Micro-CT35, SCANCO Medical AG, Brüttisellen, Switzerland) with an 18.5 µm voxel size (equivalent to slice thickness) at 70 kVp voltage and 114 µA current to measure the mineral density value (mgHA/cm3) at the ROI in each selected cross-section slice from the lesion surface to the dental pulp. To determine the depth of silver ion penetration, the average mineral density in a 1x1 mm area of each scanned slice along the scan axis from the surface lesion to normal dentin was compared (25) .

**Figure 1:**
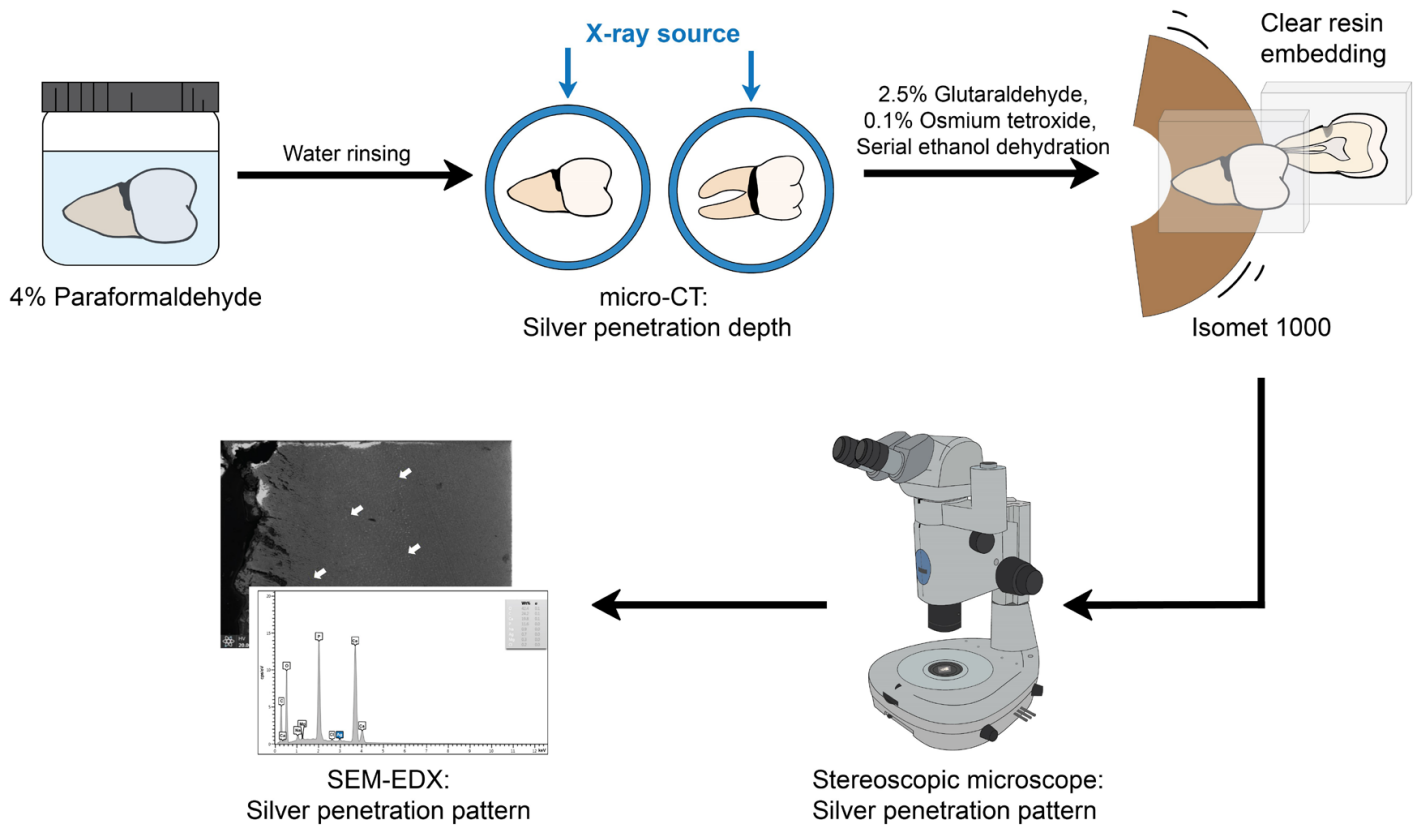
Illustration of the laboratory examination for extracted tooth samples: Micro-computed tomography (micro-CT), Stereoscopic microscope, and Scanning electron microscope with Energy Dispersive X-ray Spectrometer (FE-SEM/EDX).

Following this, each sample was remounted and rescanned, with the lesion surface facing the micro-CT x-ray source to illustrate the silver ion penetration into cementum and root surface. The scanned images were then analyzed and reconstructed with CT-Analyser software (Micro-CT35, SCANCO Medical AG, Brüttisellen, Switzerland) (Fig. 1).

Stereoscopic microscope:

Following the micro-CT analysis, teeth samples were fixed with 2.5% glutaraldehyde for 2 hours at 4°c, followed with 0.1% osmium tetroxide solution for 2 hours at 4°c. Each sample was then dehydrated in ascending ethanol series (50%, 70%, 80%, 90%, 95% for 25 minutes in each concentration; twice in 100% for 25 minutes each). After that, the teeth were embedded with epoxy resin and section vertically through the lesion’s center with Isomet 1000 (Buehler, ISOMET 1000, Buehler Ltd., Lake Bluff, Illinois, USA). The specimens were imaged under stereoscopic microscope (Nikon, smz1500, Nikon Instech Co., Ltd., Tokyo, Japan) at 2x magnification to describe silver ion penetration pattern.

Scanning electron microscope (SEM-EDX):

Following the stereoscopic microscope observation, the teeth specimens were examined using FE-SEM (FEI, Apreo, Eindhoven, Netherlands) without coating in backscattered electron mode with 20 kV at 100x, 300x, and 500x magnifications to describe silver penetration pattern. The elemental spectrum of silver particles was determined by using EDX mapping.

-Statistical analysis

Study characteristics e.g., oral health behaviors and clinical characteristics e.g., color, appearance, and surface hardness of root caries lesion at baseline and 2 weeks after silver diamine application were summarized using descriptive statistics. The change in mineral density from the surface lesions to the dental pulp and normal dentin at the other side were plotted using line graph. The Wilcoxson signed rank test statistic was used to compare the change in the number of each bacterial strain (STATA MP version 16).

## Results

Ten participants aged 60.7±6.5 years old, with a total of 16 teeth, were included in the study. The majority of them reported brushing at least twice daily (n = 8) with fluoride toothpaste (n =9). After SDF application for 2 weeks, the following favorable improvement were observed: the samples were darker in color, predominantly turning black (15 teeth, 93.8%), had increased surface hardness (7 teeth, 43.8%), had improved sensitivity (non-sensitive teeth, 7 teeth, 43.8%), and were negative to air blowing (5 teeth, 31.2%) ( Table 2). Furthermore, of 10 study participants, only one subject reported metallic taste and unpleasant smell and two subjects reported mild irritation symptom.

**Figure 2:**
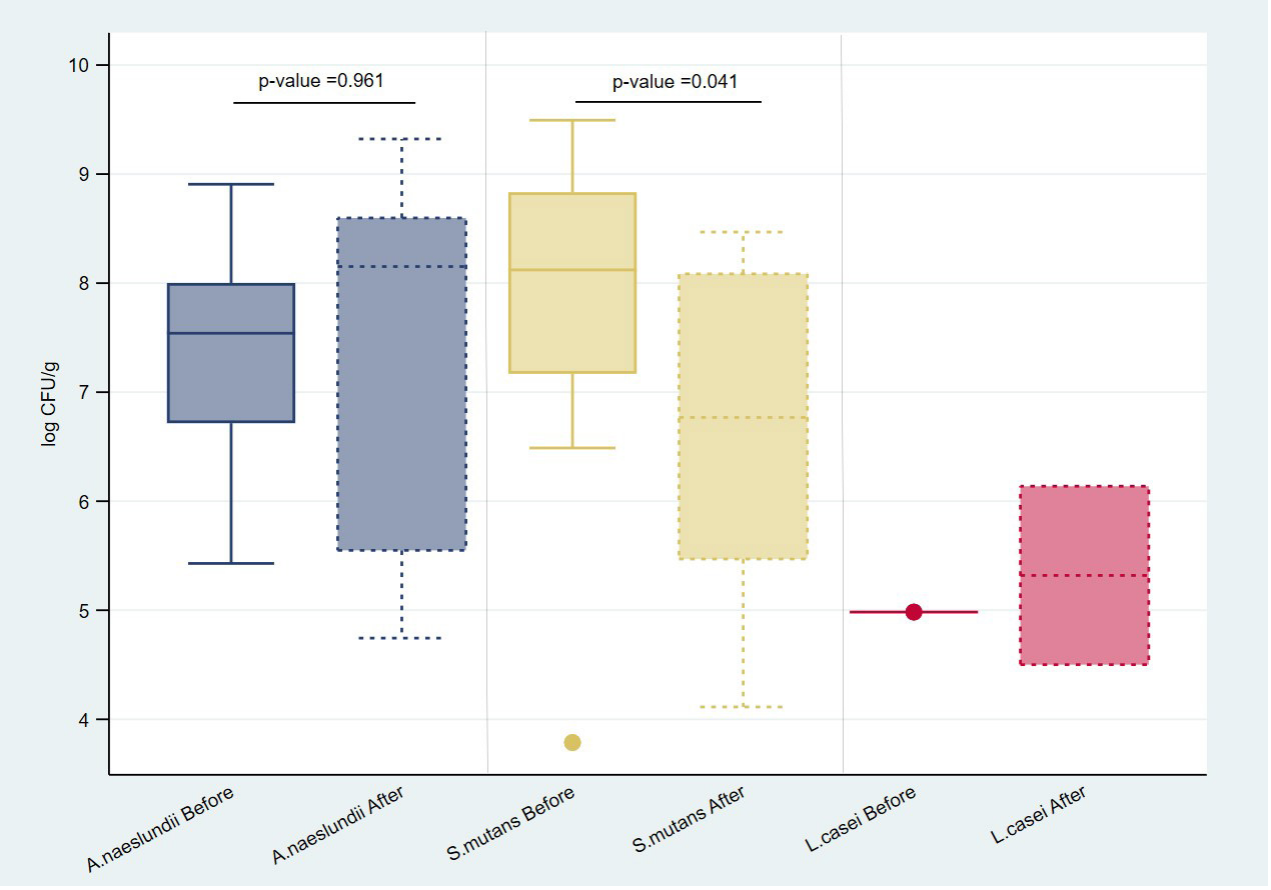
Log CFU/g of *A. naeslundii*, *S. mutans*, and *L. casei* from root carious lesions at baseline and 2 weeks after 38% SDF application.

Only *S. mutans* had a significantly lower number of bacteria (log CFU/g) after 2 weeks of SDF application (*p* = 0.041). There was an increase in the number of *A. naeslundii* but was no statistically significant difference (*p* = 0.961) (Fig. 2). At baseline and follow-up, *L. casei* was found in just one sample and two samples, respectively.

**Figure 3:**
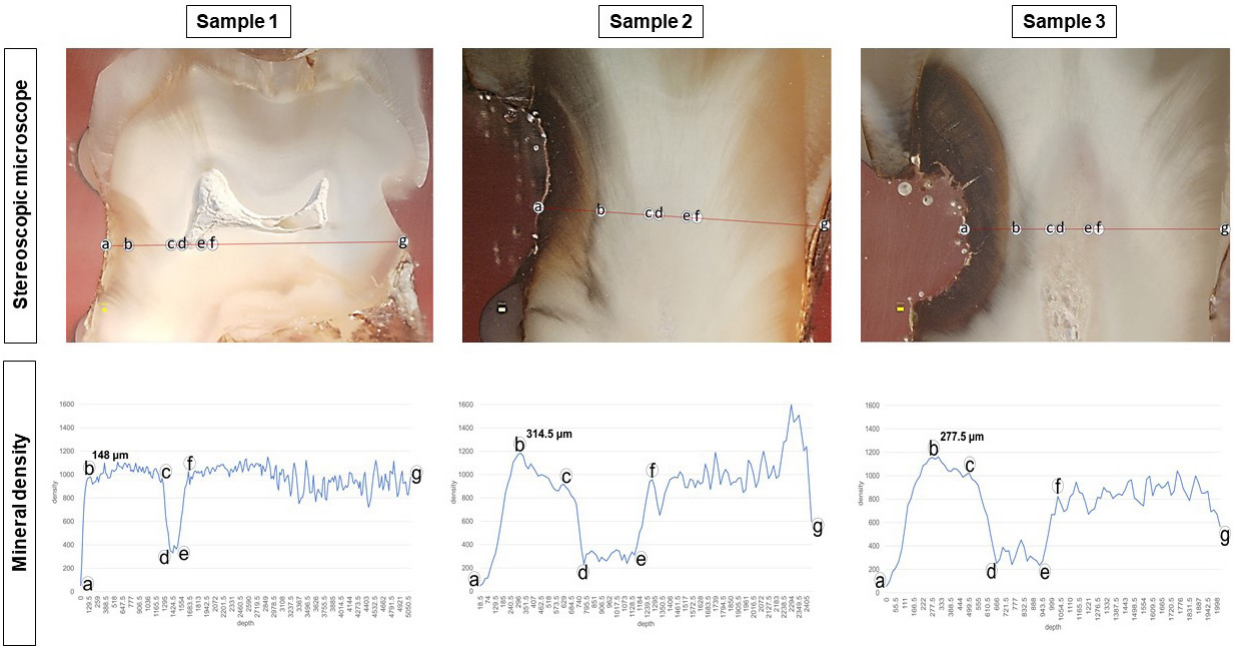
Cross-sectional stereoscopic microscope images of root carious lesions (sample 1, 2, and 3) treated with 38% SDF and corresponding mineral density (mgHA/cm3) and depth interval from the lesion surface (a) toward the opposite side (g). A stereoscopic microscope indicated that silver penetration resulted in dark band development throughout the lesions, along the dentinal tubule (a to b). The penetration of silver increased the root carious lesion density, which was correlated with its lesion depth, with a range of 148-314 µm (b).

**Figure 4:**
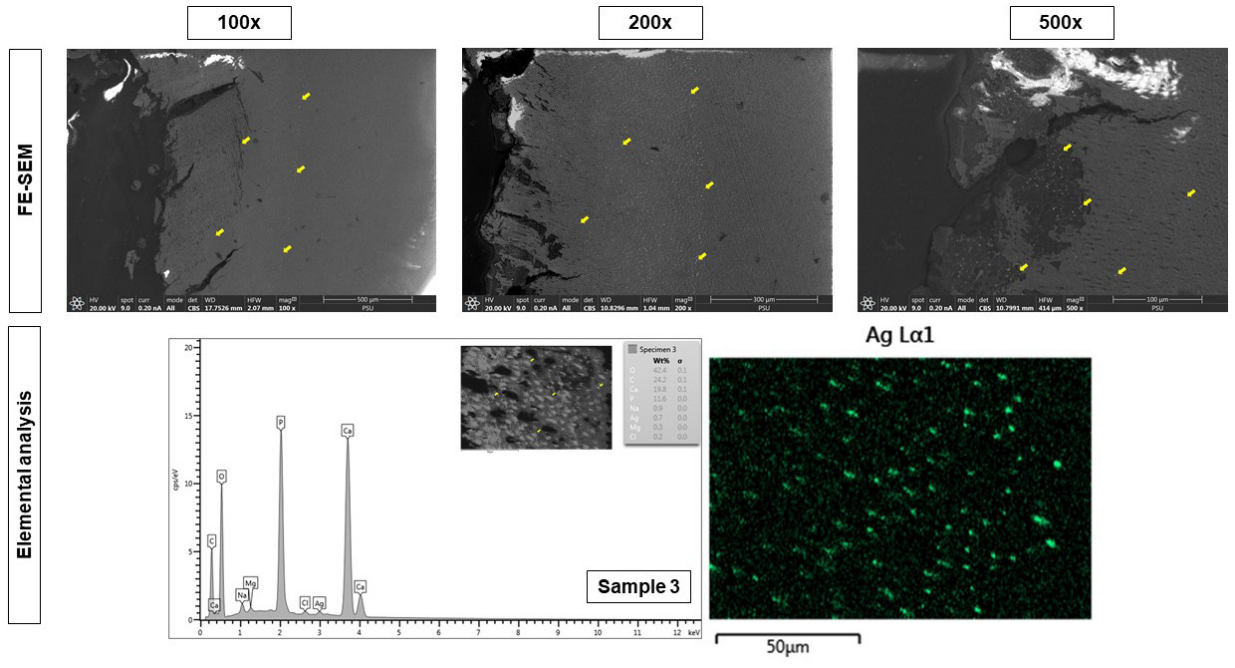
FE-SEM micrographs at 100x, 200x, and 500x magnification and EDX mapping analysis of root carious lesions treated with 38% SDF of sample 3. The bright particles distributed throughout the carious lesions. The corresponding EDS mapping and point analysis on the bright particles indicated the presence of silver element.

Micro-CT analysis revealed that the penetration of silver increased the density of root carious lesion, which was correlated with its lesion depth, with the range of 148-314 µm. A stereoscopic microscope examination showed that silver penetration resulted in dark bands appearing along the dentinal tubule to the direction of the dental pulp (Fig. 3). An FE-SEM analysis of the pattern and location of mineral deposition within lesions showed that silver ions were observed to be densely deposited on the surface of the lesions and penetrated through the dentinal tubule into the dental pulp direction related to the cavity depth. According to the EDX mapping study, the increased density was related to silver (Fig. 4).

## Discussion

Based on clinical, microbiological, and morphological profiles, this study supported non-restorative cavity control with SDF is an alternative for treating cavitated root carious lesions.

Although different disease-controlling measures for root caries, including application of fluorides vanish, chlorhexidine mouthwash, antimicrobials agents, salivary enhancers, and patient education have been tested (9,10). SDF has gained attention in recent years for its effectiveness against caries, particularly for arresting dentine caries not just in primary teeth but also in root caries (8,10). Annual SDF treatment has a strong evidence in the literature to prevent and treat root caries (9,10,26,27). In contrast, a recent randomized clinical trial study among nursing home older adults showed that a single SDF application had no significant additional preventive effect on root caries lesions at 1-year follow-up. That might be explained by that most participants in that study received regular preventive care, low caries progression rate, and selection bias. Participants in that study had an average of four dental hygienist visits, including professional cleaning and fluoride varnish application, and an average of two dentist visits for operative treatment (28). Contrary to our study that conducted among community-dwelling older adults, although they were able to perform oral hygiene care, but the cleanliness efficiency was questionable, and they were more likely to be problem-oriented attendees. Furthermore, previous epidemiologic studies in this area among adults aged 35-65 years revealed a high prevalence of periodontal disease (26.8%), root caries (21.3%) (29), and sugary snack consumption (29.8%) (30), emphasizing the importance of implementing effective noninvasive measures to reduce the burden of untreated root caries in this population.

SDF is a colorless, odorless solution of silver, fluoride, and ammonium ions, with the ammonia acting as a solution stabilizer. SDF (38%) has a high concentration of fluoride ions (44,800 ppm) and silver ions (255,000 ppm). When applied on carious tooth tissue, a series of chemical reactions from silver and fluoride ions promote tooth desensitization by dentinal tubule blockage, and carious lesion arrest by bacterial death, remineralization of demineralized tooth and inhibition of dentinal collagen degradation. However, these chemical reactions result in staining carious lesions, whereas sound enamel does not stain (8). To assess the success of SDF application, lesion color was traditionally used to evaluate whether a lesion was arrested. However, rather than its appearance, this is more properly determined by how the lesion feels when a ball-ended probe is dragged across it (8,20). The American Academy of Pediatric Dentistry (AAPD) recommends a follow-up after 2-4 weeks to evaluate the caries arrest following SDF treatment. If the lesion does not appear dark and firm on probing, reapplication is indicated (31). In our study with a 2-week follow-up, most lesions showed clinical signs of arresting root caries: darker in color and increased surface hardness when probing. However, only half of lesions (56%) showed no plaque deposit at the follow-up. This might suggest the SDF reapplication if the participants were unable to properly remove the biofilm to modify the environment to promote caries arresting. Furthermore, those study participants who still experienced dental hypersensitivity (self-reported =12.5%and testing with air blow = 25%) may benefit from SDF reapplication.

Bactericidal activity of SDF, which is the immediate action of reducing bacterial load and hence preventing bacterial re-colonization and proliferation, is well documented (12,14,15,32,33). It plays a vital role in biofilm disruption and caries protection throughout the mouth due to the ‘zombie effect,’ in which living bacteria are killed when they come into contact with silver-affected bacteria. The killed bacteria serve as an efficient reservoir of lethal metallic cations, which are continuously release to attack the living bacteria (34). In a recent review (33) has reported that *in vitro* studies using monospecies bacteria on dentin or enamel samples, SDF could prevent bacterial adhesion to the tooth surface and inhibited the growth of cariogenic bacteria and **Candida* albicans*. However, these *in vitro* testing did not fully replicate the complex biofilm ecology in human mouth and there were just a few clinical studies on the microbiological profiles of SDF-treated lesions (15-17). Our *in vivo* study analyzed plaque samples using RT-PCR before and after SDF application found that only the primary colonizer, *S. mutans*, was reduced among the three bacterial species examined. In contrast to a previous microbiome study in adults using plaque samples from root or cervical caries lesions discovered that SDF inhibited the growth of periodontitis bacteria, with *Actinomyces* spp. showing the highest reduction. Some bacterial species that produce acid tended to decline, contributing to caries arrest. There was also no significant difference in bacterial composition between samples before and after SDF administration (17). Another clinical investigation in children based on metageonic sequencing analysis has reported a decrease in *S. mutans* and *Lactobacillus* spp. in arrested caries after SDF treatment, without no significant loss of bacterial species diversity (16). A recent investigation on the alteration of microbial composition of plaque samples on SDF-treated caries in children also found no overall microbiome changes in the caries arrested by SDF, but caries-related species were reduced in arrested caries (15).

In this study, we failed to detect *L. casei* in most samples (i.e., one sample at baseline and two samples at follow-up). This may be explained by the limitation of PCR technique in which the target specificity of any RT-PCR assay is determined by the design of the primers. Perhaps, *L.casei* may not be the dominant *Lactobacillus* spp. in root carious lesions among this study group, or they may exist below the detection limit of RT-PCR. Furthermore, the colonization of *Lactobacillus* spp. requires a retentive niche that permits *Lactobacilli* spp.to accumulate, resulting in a low pH and anaerobic environment, combined with access to source of carbohydrates (35). The majority of lesions in our study were supragingival and likely to be broad shallow saucer-shaped cavities rather than deeper defined cavities. That may reduce the likelihood for being mechanically retentive site for *Lactobacilli* spp.

In our study protocol, the necrotic dentin was not removed. However, FE-SEM and micro-CT demonstrated that SDF particles were extensively deposited throughout the lesions, corresponding to dark bands observed in stereoscopic microscope. This agreed with a previous study. They found that leaving necrotic dentin did not impair silver penetration (25,36), and that silver penetration depth was dependent on the quantity of remaining dentin thickness and dentin characteristics (25). However, compared to earlier *in vitro* studies using extracted teeth, our *in vivo* investigation, the range of silver penetration depth was lesser (148-314 µm) and silver particle did not precipitate in the pulp. The penetration depth in deep carious lesion in permanent teeth was 629–2516 µm (25) and primary teeth was 17-2490 µm (36). Moreover, both studies observed silver precipitation reaching the pulp chamber (25,36). In addition to the difference in lesion features (25) and structures and mineral contents of deciduous teeth and permanent teeth (36), the lack of pulpal pressure *in vitro* tests was a plausible explanation.

There are some strength and limitations to this study. This study was undertaken during the Covid-19 pandemic, when dental practices were restricted to emergency dental treatment in order to reduce the spread of Covid-19. To our knowledge, this is the first *in vivo* study to assess the efficacy of non-restorative root caries control with SDF in clinical evidence, microbiology, and the degree of sliver precipitation. RT-PCR although is widely applied to quantify the abundance of microbial community due to its high sensitivity, RT-PCR method cannot distinguish between vital and non-vital microbes. In addition, due to its high cost, this study examined only the short-term effects of three major root-caries related bacteria. Lastly, only three samples that met the extraction criteria were examined for silver penetration. To validate silver penetration with varying residual dentin thickness, more research with a larger sample size is required.

## Conclusions

This study confirmed that SDF is effective for controlling root caries based on clinical and microbiological profiles, particularly *S.mutans* reduction. The application of SDF on root caries without removing necrotic tissue had no effect on its deep penetration into the lesion.

## Figures and Tables

**Table 1:** RT-PCR primers of each target genes and species.

Species	Genes	Primer sequence (5'-3')	Annealing temperature (°C)	Amplicon size (bp)	Reference
S.mutans (DMST 41283)	glucosyltransferase genes (gtfB)	Forward: ACTACACTTTCGGGTGGCTTGG Reverse: CAGTATAAGCGCCAGTTTCATC	58	516	Oho et al., 2000 (22)
L.casei (TISTR 1463)	DNA-dependent RNA polymerase beta-subunit gene (rpoB)	Forward: CTGCTGCTGACATCGCCGCTCGTA Reverse: TCCGCTCGCGCCACCTCTCGTTA	62	144	Park et al., 2013 (23)
A.naeslundii (TISTR 2426)	16s robosomal DNA (16s rDNA)	Forward: GCACCGAGATTCAACATGG Reverse: GGTTCTTGGATYTATGCGGTATTAG	58	117	Byun et al., 2004 (24)

**Table 2:** Clinical characteristic of root caries lesions at baseline and 2 weeks after silver diamine application.

Clinical characteristics	Baseline	2-week follow-up	N (%)
Visible plaque deposit*			
	Score 0	Score 0	6 (37.5)
	Score 1	Score 0	3 (18.8)
	Score 0	Score 1	2 (12.5)
	Score 1	Score 1	5 (31.2)
Color	Yellow	Dark brown	1 (6.2)
	Light brown	Black	5 (31.3)
	Dark brown	Black	8 (50.0)
	Black	Black	2 (12.5)
Surface hardness	Soft/ leathery	Soft/ leathery	5 (31.2)
	Soft/ leathery	Hard	7 (43.8)
	Hard	Hard	4 (25.0)
Sensitivity to air blow	No	No	7 (43.8)
	Yes	No	5 (31.2)
	Yes	Yes	4 (25.0)
Sensitivity complaint	No	No	7 (43.8)
	Yes	No	7 (43.8)
	Yes	Yes	2 (12.5)

*Visible plaque deposit was determined using visible plaque index: Score 0 = no plaque detected; Score 1 = visible plaque detected

## Data Availability

The datasets used and/or analyzed during the current study are available from the corresponding author.
